# Molecular and Functional Characterization of ssDNA Aptamers that Specifically Bind *Leishmania infantum* PABP

**DOI:** 10.1371/journal.pone.0140048

**Published:** 2015-10-12

**Authors:** Natalia Guerra-Pérez, Edurne Ramos, Marta García-Hernández, Celia Pinto, Manuel Soto, M. Elena Martín, Víctor M. González

**Affiliations:** 1 Laboratory of aptamers, Servicio de Bioquímica-Investigación, IRYCIS-Hospital Ramón y Cajal, Madrid, Spain; 2 Aptus Biotech SL, Parque Científico de Madrid, Campus de Cantoblanco, Madrid, Spain; 3 Departamento de Biología Molecular, Centro de Biología Molecular Severo Ochoa (CSIC-UAM), Universidad Autónoma de Madrid, Madrid, Spain; Royal Tropical Institute, NETHERLANDS

## Abstract

**Summary:**

A poly (A)-binding protein from *Leishmania infantum* (LiPABP) has been recently cloned and characterized in our laboratory. Although this protein shows a very high homology with PABPs from other eukaryotic organisms including mammals and other parasites, exist divergences along the sequence that convert them in potential diagnostic markers and/or therapeutics targets. Aptamers are oligonucleotide ligands that are selected *in vitro* by their affinity and specificity for the target as a consequence of the particular tertiary structure that they are able to acquire depending on their sequence. Development of high-affinity molecules with the ability to recognize specifically *Leishmania* proteins is essential for the progress of this kind of study.

**Results:**

We have selected a ssDNA aptamer population against a recombinant 6xHIS–LiPABP protein (rLiPABP) that is able to recognize the target with a low Kd. Cloning, sequencing and *in silico* analysis of the aptamers obtained from the population yielded three aptamers (ApPABP#3, ApPABP#7 and ApPABP#11) that significantly bound to PABP with higher affinity than the naïve population. These aptamers were analyzed by ELONA and slot blot to establish affinity and specificity for rLiPABP. Results demonstrated that the three aptamers have high affinity and specificity for the target and that they are able to detect an endogenous LiPABP (eLiPABP) protein amount corresponding to 2500 *L*. *infantum* promastigotes in a significant manner. The functional analysis of the aptamers also revealed that ApPABP#11 disrupts the binding of both Myc-LiPABP and eLiPABP to poly (A) *in vitro*. On the other hand, these aptamers are able to bind and purify LiPABP from complex mixes.

**Conclusion:**

Results presented here demonstrate that aptamers represent new reagents for characterization of LiPABP and that they can affect LiPABP activity. At this respect, the use of these aptamers as therapeutic tool affecting the physiological role of PABP has to be analyzed.

## Introduction

Leishmaniasis is caused by *Leishmania* spp. and is transmitted by sand flies. The disease, also known as kala-azar (black fever) or Assam fever, has a wide distribution that extends from the Mediterranean to Middle Asia, to southern Russia and China [1, 2]. Diagnosis of leishmaniasis is routinely performed by finding the parasite in smears from skin lesions or in bone marrow, spleen, liver, or blood smears using microscopic examination. However, serological, immunological and PCR-based methods are being developed [3].

*Leishmania* is a parasitic protozoan of the trypanosomatids family that possesses a digenetic life cycle with two discrete morphological phases: the promastigote, which develops extracelullarly within the gut of the insect vector, and the amastigote that is specialized to survive within the macrophage’s phagolysosome of the vertebrate host. Thus, it is assumed that trypanosomatids parasites need a regulated expression of stage-specific genes to survive extreme environmental changes in which poly (A)-binding protein (PABP) regulation could play some role. Different PABPs have been described in *T*. *cruzi* (TcPABP1) [4], *T*. *brucei* (TbPABP) [5], *L*. *major* PABP (LmPABP1, LmPABP2 y LmPABP3) [6, 7] or *L*. *amazonensis* (LaPABP) [8]. Very recently, we have described the first PABP homologue from *L*. *infantum*, called LiPABP, which conserves the main domains present in other PABPs and maintains poly(A) binding properties [9].

The PABP of eukaryotes binds the 3´ end poly (A) tail of the mRNAs and participates in different cellular processes such as translational initiation and termination and in mRNA turnover [10–12]. When bound to the poly (A) tail, PABP circularizes mRNA molecules via its interaction with translational initiation factors at the cap, enhancing translational initiation and stabilizing mRNA [10–12]. In addition, interaction with the release factor eRF3 and dissociation of PABP from the poly (A) tail is necessary for progressive deadenylation and consequent decay for many transcripts [13–16]. Furthermore, this protein seems to be involved in mRNA transport from the nucleus to the cytoplasm [12, 17].

In our laboratory, we are very interested to isolate and characterize aptamers against *Leishmania* proteins in an attempt to develop diagnostic and/or therapeutic tools against leishmaniasis. At this respect aptamers targeting *L*. *infantum* KMP-11 [18, 19], LiH2A [20, 21] and LiH3 [22] have been selected and characterized in our laboratory.

Aptamers are structured polynucleotide sequences isolated from randomized oligonucleotide libraries by systematic evolution of ligands by exponential enrichment (SELEX) technology, that selectively bind target molecules with high affinity and specificity [23–25]. Aptamers are able to form stable and specific complexes with the targets that have dissociation constants in the nanomolar range due to the highly defined tertiary structures that are able to adopt depending on their sequence, and can clearly distinguish between even closely related protein targets [26, 27]. Aptamers have several advantages over antibodies because of the nature of nucleic acids such as increased stability, easy regeneration and simple modifications with different reporters during their synthesis. In addition, they are significantly smaller, can be isolated rapidly *in vitro* and do not elicit a significant immune response [28, 29].

Aptamers are also selected against defined protein target in order to use them as molecular tools to study the interaction with other molecular partners or to identify their sites of action. Indeed, aptamers have been generated against transcription factors and shown to interfere with a range of molecular interactions both *in vitro* and *in vivo* [30, 31]. Moreover, further functional analysis of LiPABP requires molecules that specifically bind the protein target in order to affect PABP-poly (A) interaction and aptamers compete to antibodies in this purpose. In addition, molecules recognizing LiPABP might be very important as detection, diagnostic or therapeutic tools.

In this paper, we have used SELEX to generate a DNA aptamer population that binds LiPABP with high affinity. In addition, we detail the isolation and characterization of three aptamers that are able to recognize specifically the protein with affinity in the low nanomolar range. These aptamers detect LiPABP from 2500 *L*. *infantum* promastigotes in a significant manner and, in consequence, they could be used in the development of diagnostics systems for leishmaniasis. Furthermore, analysis of selected aptamers reveals that one of them, ApPABP#11, disrupts the binding of LiPABP to poly (A). This ability of this aptamer may be used in regulating the function of LiPABP *in vivo*. In addition, we demonstrate that the three aptamers are able to bind and purify LiPABP from lysates of cells overexpressing the protein. This capacity would be useful for further characterization of the LiPABP activity.

## Materials and Methods

### Materials

Products were obtained from Sigma-Aldrich (Spain) except those indicated in the text. RND40 starting population, individuals DNA aptamers, and their derivatives were purchased from IBA GmbH (Göttingen, Germany).

### Cell culture and extract preparation

*Leishmania infantum* (MCAN/ES/96/BCN150) promastigotes were grown at 26°C in RPMI 1640 medium (PAA Laboratories), supplemented with 10% (v/v) foetal calf serum (PAA Laboratories), 10 U/mL penicillin and 100 μg/mL streptomycin (Gibco). Cultures containing *L*. *infantum* promastigotes were pelleted and lysed in ice-cold RIPA buffer (50 mM Tris-HCl pH 7.6, 150 mM NaCl, 1 mM EDTA, 1% NP-40, 0.5% sodium deoxycholate, 0.1% SDS) and centrifugated at 17000 x g for 20 min, to obtain total lysates. Protein determination was performed by the method of Bradford [32]. The supernatant volume was accurate measured to calculate that corresponding to 10^3^ *L*. *infantum* promastigotes.

Transient transfections with pcDNA3-Myc-LiPABP or pcDNA3-Flag-LiPABP were essentially performed as described previously [33]. After 24 h of transfection, the medium was removed and the cells washed twice with ice-cold buffer A (20 mM Tris-HCl pH 7.6, 1 mM DTT, 1 mM EDTA, 1 mM PMSF, 1 mM benzamidine, 2 mM sodium molybdate, 2 mM sodium β-glycerophosphate, 0.2 mM sodium orthovanadate, 120 mM KCl, 10 μg/mL antipaine, 1 μg/mL leupeptin, 1 μg/mL pepstatin) and lysed in the same buffer containing 1% Triton X-100 (50 μL/10^6^ cells). Cell lysates were centrifuged at 12000 x g for 15 min and the supernatants were kept at -80°C until used. Protein determination was performed as above.

### Expression and purification of recombinant *L*. *infantum* proteins

*L*. *infantum* PABP protein (Mw = 65 kDa) was cloned in the pQe30 expression vector and the recombinant HIS-LiPABP (rLiPABP) was purified by affinity chromatography on Ni-NTA resin columns (QIAGEN) as described [34]. Briefly, the cells expressing rLiPABP were harvested and resuspended in binding buffer (20 mM Tris HCl pH 7.8, 0.5 M NaCl, 8 M urea) supplemented with 5 mM Imidazol, at 2–5 volumes x g-1 of wet weight. After binding to a Ni-nitroacetic acid column recombinant proteins were gradually refolded on the affinity column as described [35]. Afterwards, rLiPABP was eluted with 0.3 M imidazole and stored at -80°C.

Other HIS-tagged *Leishmania* proteins used in this study were purified in a similar way (unpublished data). Eukaryotic eIF2 protein was purified from calf cerebral cortex as previously described [36].

### *In vitro* selection

Selection of DNA aptamers for recombinant *L*. *infantum* PABP (rLiPABP) was performed as described previously [20]. In brief, ssDNA oligonucleotides containing a central randomized region of 40 nucleotides flanked by two conserved 18-nucleotides regions in each end (RND40, 5’-GCGGATGAAGACTGGTCT-40N-GTTGCTCGTATTTAGGGC-3’) (IBA GmbH, Germany) were denatured at 90°C for 10 minutes and then cooled on ice for 10 min. For the initial SELEX round, 50 μg of RND40 (Mw = 25 kDa) were mixed with 2 μg of rLiPABP in 200 μL of selection buffer (20 mM Tris-HCl pH 7.4, 1 mM MgCl_2_, 150 mM NaCl, 5 mM KCl, 0.2% BSA) and incubated 26°C for 30 minutes. The bound aptamer-rLiPABP complexes were purified by adding 20 μL of Ni-NTA superflow (Qiagen) for 1 h at 4°C. After washing three times with 1 mL of selection buffer, the ssDNA-protein complexes were resuspended in 20 μL of distilled H_2_O and amplified by 15 cycles of PCR using the primers named F3 (5´ GCGGATGAAGACTGGTGT 3´) and R3 (5´ GTTGCTCGTATTTAGGGC 3´) under the conditions of 1 μM/primer, 250 μM dNTPs, 2 mM MgCl_2_ and 1.25 U Taq polymerase in a final volume of 50 μL. PCR product was ethanol-precipitated. After 4 rounds, the pool named SEL4LiPABP was amplified and labeled by PCR using digoxigenin-labeled primer F3 and non-labeled primer R3 for further analysis. Both non-labeled and labeled primers were obtained from IBA GmbH (Germany).

### Aptamer cloning and sequencing and secondary ssDNA structure prediction

Aptamers were amplified by using 1U/50 μL Taq DNA polymerase (Biotools) in a reaction also containing 1x PCR buffer, 1.25mM MgCl_2_, 125 μM dNTPs, 1 μM primer 1 and 1 μM primer 2. The dsDNA product with ‘A’-overhangs was cloned into pGEM-T Easy-cloning vector (Promega) following manufacturer’s instructions. Individual clones were sequenced by Sanger method using T7 (5´-TAATACGACTCACTATAGGG-3´) and Sp6 primers (5´- ATTTAGGTGACACTATAGAA-3´) (IBA GmbH, Germany).

Selected ssDNA molecules were subjected to secondary structure prediction using the program Rapidshare [37], to fold DNA sequences into their minimum free energy (MFE) secondary structure, as well as to compare the obtained structures.

### Enzyme-Linked OligoNucleotide Assay (ELONA)

ELONA was used to analyze the affinity of the aptamers for the target as in Ramos et al., 2007 [20]. First, in order to assess the enrichment of the selected population, rLiPABP was diluted to 2.5 μg/mL concentration in selection buffer and 200 μL of the solution were incubated in a 96-well microtiter plate (NUNC) overnight at 4°C and, then, washed four times in selection buffer. Afterwards, digoxigenin-labeled SELLiPABP aptamer population or digoxigenin-labeled RND40 library were diluted in selection buffer at different concentrations (0–80 nM), denatured for 10 min at 95°C and then cooled for 10 min on ice. Next, 200 μL of the solution were added to each well, the plate incubated at 1 h at 26°C and then washed four times with selection buffer to remove unbound ssDNA. Afterwards, 200 μL of a 1:1000 dilution of anti-digoxigenin antibody conjugated with horse-radish peroxidase (POD) (Roche) was added to the individual wells. Following 1 h incubation at room temperature on a shaking platform, the plates were washed four times and developed using ABTS solution (Boehringer–Mannheim) according to the manufacturer’s instruction. OD values at 405 nm were determined using a microplate reader from TECAN.

In order to determinate the affinity constant of the selected populations or the individual aptamers to rLiPABP, the protein was plated as above and afterwards, incubated with the digoxigenin-labeled SELLiPABP aptamer population or the individual aptamers at concentration indicated in the figure legends, denatured for 10 min at 95°C and cooled for 10 min on ice, and then incubated at 26°C for 1 h. Next, 200 μL of a 1:1000 dilution of anti-digoxigenin antibody conjugated with POD were added to the individual wells and developed as above.

In other set of experiments, rLiPABP was plated to the required concentrations (0–5 μg/well) and incubated with 200 μL of digoxigenin-labeled SELLiPABP diluted in selection buffer at 80 nM concentration and the ELONA developed as above. To assess the specificity of the aptamers, similar experiments were performed coating the wells with 2 μg of LiPABP, other recombinant *Leishmania* proteins (LiIF2α, LiIF2β, LiIF2γ, LiH2A, LiP2a) or eukaryotic eIF2 protein, which were incubated with 200 μL of digoxigenin-labeled SELLiPABP aptamer populations at 40 nM as above.

To study the sensitivity of the aptamers to recognize LiPABP from *L*. *infantum* promastigotes, lysates corresponding to increasing number of parasites (0–5000) were plated in coating solution (KPL) and incubated with digoxigen-labeled aptamers at 10 nM, and the ELONA was developed as above.

### Slot blot analysis with aptamers

Several amounts of rLiPABP (0–25 ng) and 25 ng of BSA, as negative control, were transferred onto nitrocellulose membranes under vacuum. Filter were washed three times in PBS-T (10 mM sodium phosphate, 0.15 M NaCl, 0.05% Tween-20, pH 7.5) for 10 min and then blocked with 5% milk in PBS-T for 1 h at room temperature. Afterwards, membranes were incubated with different concentrations (50–400 nM) of digoxigenin-labeled SELLiPABP aptamer population or 50 nM of individual aptamers in selection buffer for 1 h at room temperature. Next, membranes were washed with selection buffer three times and probed with anti-digoxigenin-POD antibody diluted 1:1000, for 1 h at room temperature. Excess enzyme was removed by three subsequent washes with selection buffer. Finally, the membranes were developed with enhanced chemiluminescence’s kits (GE Healthcare) and exposed to hyperfilm.

### Poly (A)-sepharose binding assay

To test the ability of individual aptamers to specifically block LiPABP-poly (A) binding, poly (A) binding experiments using poly (A)-sepharose microparticles were carried out as described previously [9]. Briefly, lysates from *L*. *infantum* promastigotes (100 μg) or 25 μg of lysates from HEK293T cells overexpressing Myc-LiPABP, in 200 μL of binding buffer (20 mM Tris-HCl, pH 7.4, 1 mM DTT, 1 mM MgCl_2_, 5 mM EDTA and 1 mM PMSF), were incubated with 50 μL of poly(A)-Sepharose (50%) for 1 h at 4°C in the absence or the presence of 400 pmoles of the selected aptamers or the naïve RND40 population. The beads were washed six times with binding buffer and proteins were eluted with Loading/Sample buffer. The membrane was soaked with blocking buffer (5% non-fat dry milk, 10 mM Tris-HCl at pH 7.5, 100 mM NaCl, 0.1% Tween-20) 1 hour at room temperature, and incubated with anti-Myc antibody (Santa Cruz Biotechnology) (to detect recombinant LiPABP expressed in HEK293T cells) or polyclonal sera from infected dogs (to detect endogenous LiPABP) overnight at 4°C. After repeated washing, the membrane was incubated with anti-mouse IgG sheep secondary antibody HRP conjugate (GE Healthcare) or anti-dog sheep secondary antibody HRP (Bethyl Laboratories, USA), respectively.

### PABP pull-down assay

To test the ability of individual aptamers to specifically bind to and purify the LiPABP presents in lysates, purification experiments using streptavidin agarose (Solulink) were carried out. Streptavidin agarose were equilibrated for 30 min at 4°C in buffer A (20 mM Tris-HCl pH 7.6, 1 mM DTT, 1 mM EDTA, 1 mM PMSF, 1 mM benzamidine, 2 mM sodium molybdate, 2 mM sodium β-glycerophosphate, 0.2 mM sodium orthovanadate, 120 mM KCl, 10 μg/mL antipaine, 1 μg/mL leupeptin, 1 μg/mL pepstatin) and 1 mg/ml of cytochrome C to block nonspecific binding. Meanwhile, 50 μg lysates from HEK293T cells overexpressing Flag-*Li*PABP were incubated with (125 pmol) of biotin-labeled aptamers for 30 min at 4°C. To carry out the purification, 30 μL of 50% streptavidin agarose were added to the mixture and incubated for 2 h at 4°C in agitation. Next, samples were centrifuged for 5 min at 3500 g at 4°C and the precipitates were washed five times with 0.5 mL of buffer A at 4°C. The retained proteins were eluted in 3x loading buffer and resolved by 10% SDS-PAGE and transferred to polyvinylidene difluoride (PVDF) membranes. The membrane was soaked with blocking buffer for 1 h at room temperature, and incubated with anti-Flag antibody (Sigma) overnight at 4°C. After repeated washing, the membrane was incubated with anti-mouse IgG sheep secondary antibody HRP conjugate (GE Healthcare).

### Statistical analysis

Data are presented as an average value ± standard error of the mean (SEM) from three to six independent measurements in separate experiments and analyzed using GraphPad Prism v4 (San Diego, CA, USA). The statistical significance was performed by analysis of variance ANOVA followed by Tukey´s or Dunnett´s test.

## Results

### Selection and characterization of aptamers against the LiPABP

The aptamer selection target used in this study was recombinant 6xHIS-LiPABP. This protein has been described and characterized in our lab showing its capability to complex with a poly (A) oligonucleotide [9]. LiPABP was purified and used as target for four rounds of *in vitro* ssDNA aptamer selection with an RND40 degenerate library as described in the Materials and Methods section. PCR reactions were restricted to 15 cycles to avoid artifacts produced when high amounts of template are used. We analyze the four rounds of selection by PCR. The results showed increasing amounts of DNA product in rounds 3 and 4, which is consistent with a higher recovery of aptamers targeting LiPABP throughout the SELEX process

**Fig 1:**
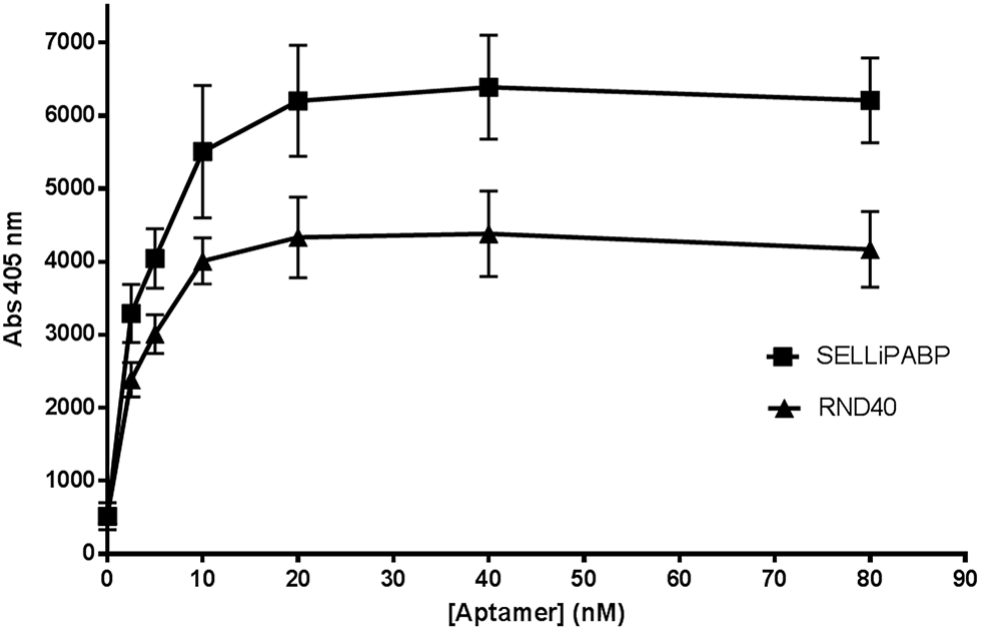
Aptamers are selected after 4 round of SELEX. The stringency of the SELEX procedure was monitored using ELONA as described in “Materials and Methods” section. SELEX pool from round 4 (SELLiPABP) and the starting round (RND40) were assayed for binding to LiPABP protein. Recombinant LiPABP was plated at 500 ng/well (7.5 pmol/well) and incubated with 200 μL of digoxigenin labeled SELLiPABP aptamer population or digoxigenin-labeled RND40 library (0–80 nM). Finally, anti-digoxigenin-POD antibodies were added plate was revealed with ABTS solution at 405 nm. All the experiments were made in triplicate and average of two different experiments is shown.

**Fig 2:**
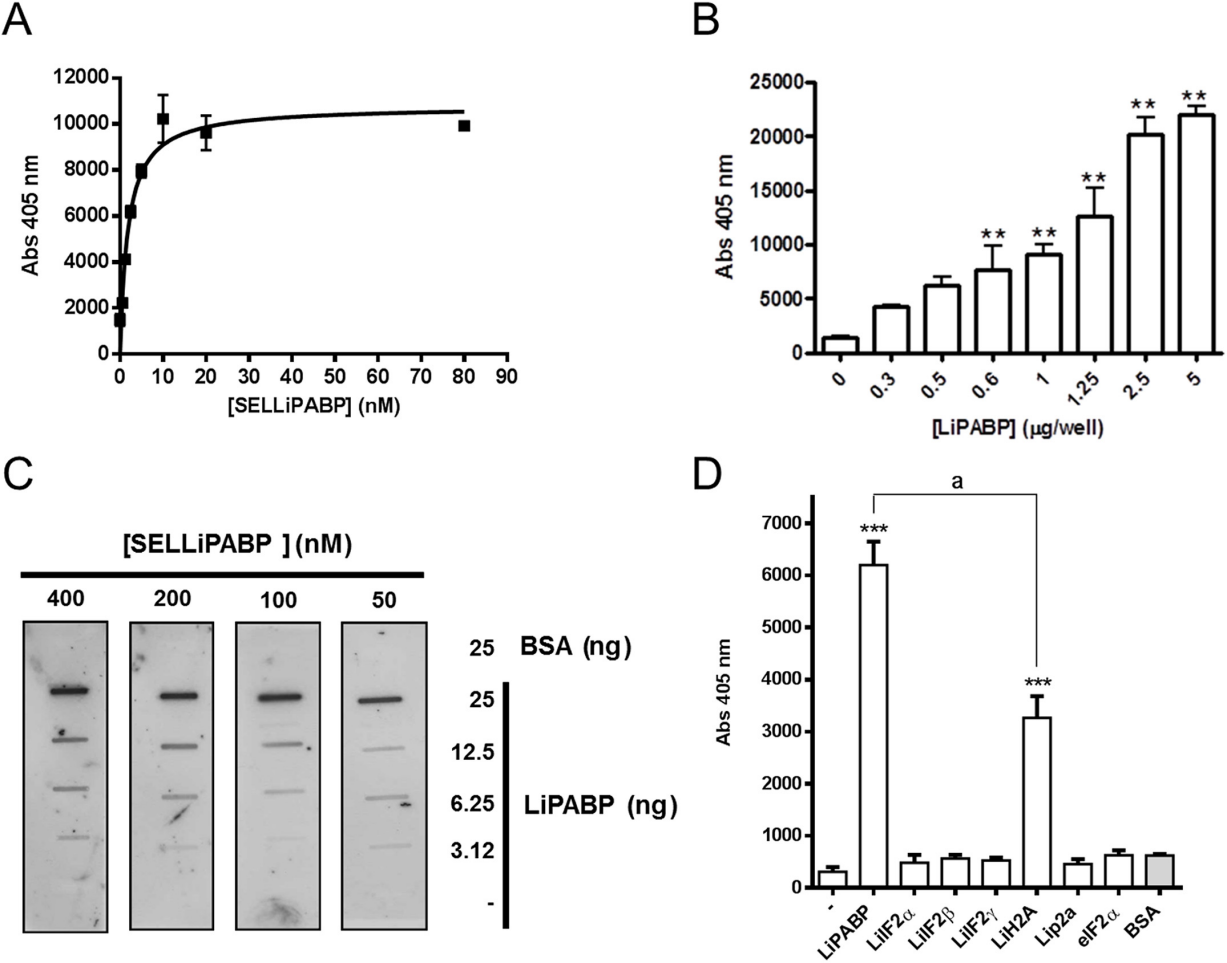
Binding capability and specificity of the SELLiPABP aptamer population for LiPABP. ELONA (**A, B** and **D**) and slot-blot (**C**) assays were performed as described in “Materials and methods” section. (**A**) Recombinant LiPABP was plated at 500 ng/well (7.5 pmol/well) and incubated with the digoxigenin-labeled SELLiPABP aptamer population at the concentrations indicated (0–80 nM). All the experiments were made in triplicate and average of four different experiments is shown. (**B**) Recombinant LiPABP was plated to several concentrations between 0–5 μg/well (0–75 pmol/well) and incubated with 80 nM of digoxigenin-labeled SELLiPABP aptamer population. All the experiments were made in triplicate and average of four different experiments is shown. Statistical significance between each concentration of LiPABP protein and the value obtained for the blank **, p<0.01. (**C**) BSA and rLIPABP at the concentration indicated were fixed under vacuum to a nitrocellulose membrane. Membranes were blotted with digoxigenin-labeled SELLiPABP aptamer population at several concentrations. Afterwards, the membrane was probed with anti-digoxigenin-POD antibodies, developed with enhanced chemiluminescence’s kits and exposed to hyperfilm. The experiment shown is representative of at least three different experiments. (**D**) Recombinant proteins LiPABP, LiIF2α, LiIF2β, LiIF2γ, LiH2A, LiP2a or eukaryotic eIF2 protein were plated at 2 μg/well and incubated with 40 nM of digoxigenin-labeled SELLiPABP aptamer population. Afterwards, anti-digoxigenin-POD antibody was added and the plates were developed using ABTS solution. All the experiments were made in triplicate and average of four different experiments is shown (a, p<0.001 versus LiPABP value; ***, p<0.001 versus value obtained for the blank).

The initial RND40 population and the selected SELLiPABP pool were then tested for its ability to bind rLiPABP using ELONA. In only four rounds of aptamer selection, SELLiPABP population displayed increased binding to rLiPABP protein compared with the initial pool suggesting that it is enriched in ssDNA sequences that recognize rLiPABP relative to the naïve RND40 population (Fig 1). Consequently, we decided to analyze the binding affinity of the SELLiPABP aptamer population to *L*. *infantum* LiPABP using ELONA and slot blot assays. Thus, in a first approach, the wells were coated with recombinant LiPABP protein and different concentrations of digoxigenin-labeled SELLiPABP were tested as described in Materials and methods section (Fig 2A). Data were analyzed using non-linear regression showing that they responds to a one-site binding curve with an equation y = (x × Bmax)/(x + Kd) where Bmax is the maximal binding and Kd is the concentration of ligand required to reach half-maximal binding. Results indicate that SELLiPABP is able to detect rLiPABP in a concentration-dependent manner with Kd = 3.87 ± 0.67 nM. In addition, an exhaustive analysis of the data represented in Fig 2A showed that a concentration so low as 2.48 nM of SELLiPABP is able to detect from 500 ng (7.5 pmol) of recombinant LiPABP in a significant manner (p<0.01).

In order to determine the sensitivity of the selected SELLiPABP population we performed an analysis in which wells were coated with quantities ranging from 0–5 μg of recombinant LiPABP and incubated in the presence of digoxigenin-labeled SELLiPABP as described in Materials and Methods section. As seen in Fig 2B, it was further confirmed that the interaction affinity among the protein and the aptamer population is directly proportional to the quantity of LiPABP. A more detailed analysis of the data indicated that SELLiPABP (80 nM) were able to significantly detect from 0.625 μg (9.5 pmol) of LiPABP (p<0.01). All the above results confirm that SELLiPABP is a suitably enriched population that could be used as a source for isolation of high affinity aptamers.

In the slot blot assays several amounts of recombinant LiPABP and BSA were transferred onto nitrocellulose membranes and the immobilized proteins were probed with four different concentrations of digoxigenin-labeled SELLiPABP as described in Materials and methods section. As shown in Fig 2C, SELLiPABP showed strong affinity to recombinant LiPABP but did not show any affinity to negative control BSA, thereby indicating their specificity towards the LiPABP antigen. In this type of experiments, as few as 6.25 ng of LiPABP were detected after incubation of the nitrocellulose membrane with 50 nM SELLiPABP.

To study the specificity of SELLiPABP we tested the binding of these aptamers against other proteins of *L*. *infantum* (histone LiH2A, the ribosomal protein LiP2a and potential translation initiation factors LiIF2α, LiIF2β and LiIF2γ) and the eukaryotic translation initiation factor (eIF) 2 by ELONA (Fig 2D). Data showed that in the assayed conditions SELLiPABP population specifically recognizes LiPABP. Thus, the signal output for LiPABP was significantly higher than for LiH2A (*P*<0.001; ANOVA followed by Tukey´s test) meanwhile the signal for the other proteins was similar to that for negative control. The recognition of LiH2A might be probably due to the basic nature of the histone and also the high amount of protein used in the assay. Additionally, western blot assays were also performed in which cytoplasmic and nuclear fractions from promastigotes of *L*. *infantum* and recombinant LiPABP protein were blotted on PVDF membranes and then probed with digoxigenin-labeled SELLiPABP. However, aptamer population was not able to detect specifically LiPABP in these assays in which the protein is previously denatured (data not shown). It is not surprising because aptamers are selected using the native PABP and, consequently, they can recognize or not the denatured protein.

### Isolation and molecular characterization of high-affinity individual aptamer sequences

In view of these results, we decided to isolate and identify aptamer sequences that bind LiPABP with high affinity from the SELLiPABP population obtained after four rounds of selection to determinate whether or not these aptamers could be used as biorecognition molecules and laboratory tools.

**Fig 3:**
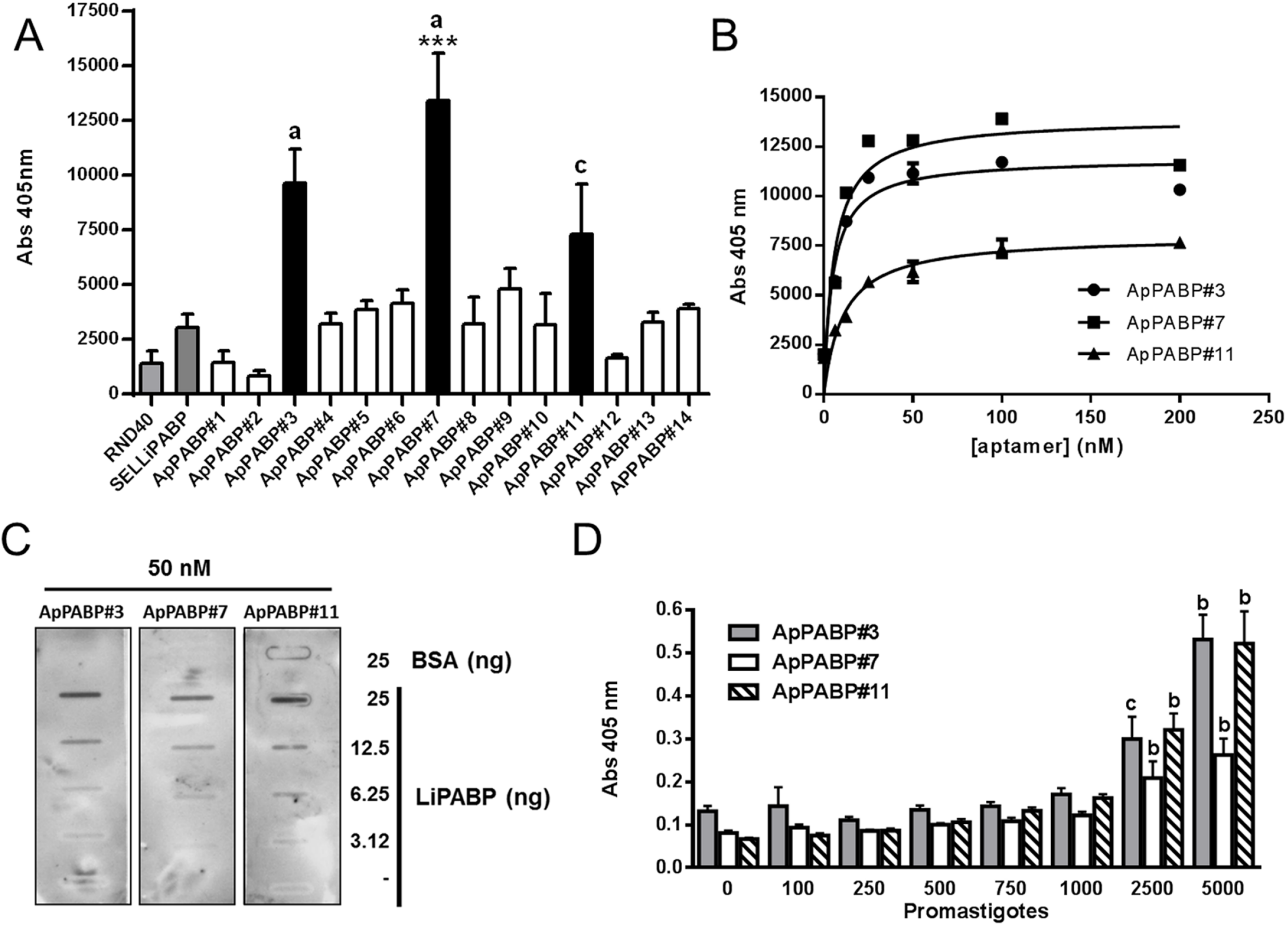
Aptamers ApPABP#3, ApPABP#7 and ApPABP#11 show a high affinity for LiPABP. (**A**) Analysis of the binding capability of the individual aptamers by ELONA. rLiPABP was plated at 1 μg/well (15 pmol/well) and incubated with the digoxigenin-labeled SELLiPABP aptamer population at 80 nM and, finally, incubated with anti-digoxigenin-POD antibodies and revealed with ABTS solution at 405 nm (a, p<0.001; c, p<0.05 versus RND40 value; ***, p<0.001 versus SELLiPABP value). (**B**) Binding affinity of ApPABP#3, ApPABP#7 and ApPABP11 to LiPABP by ELONA. Recombinant protein LiPABP was plated at 500 ng/well (7.5 pmol/well), incubated with the digoxigenin-labeled aptamers at the concentrations between 0 and 200 nM and revealed as above. All the experiments were made in triplicate and average of four different experiments is shown. (**C**) BSA and rLiPABP at the concentration indicated in the figure were fixed under vacuum to a nitrocellulose membrane. Membranes were blotted with digoxigenin-labeled ApPABP#3, ApPABP#7 and ApPABP#11 aptamers at 50 nM concentration. Afterwards, the membranes were probed with anti-digoxigenin-POD antibodies, developed with enhanced chemiluminescence’s kits and exposed to hyperfilm. The experiments shown are representative of at least three different experiments. (**D**) Sensitivity of the aptamers to recognize LiPABP from *L*. *infantum* promastigotes was analyzed by ELONA. Total lysates corresponding to increasing amounts of parasites (10^2^-5x10^3^) were plated and incubated in the presence of digoxigenin-labeled ApPABP#3, ApPABP#7 and ApPABP#11 at 10 nM and assays were performed as in Fig 1. All the experiments were made in triplicate and average of 3–4 different experiments is shown. Statistical significance was calculated between each parasite number and the value obtained for 0 parasites (b, p<0.01; c, p<0.05).

With this purpose aptamer population was cloned and sequenced, and the affinities of the individual anti-LiPABP aptamers for their target were estimated by ELONA as above. Results showed that most of the aptamers analyzed bind to LiPABP with higher affinities than RND40 control and SELLiPABP population (Fig 3A) and, moreover, that three of them, ApPABP#3, ApPABP#7 and ApPABP#11, bound to the target with affinities significantly higher than the others. No binding to an unspecific protein (BSA) occurred under these conditions (data not shown).

In order to calculate the dissociation constant of the selected aptamers, ELONA assays were carried out as above. As shown in Fig 3B, ApPABP#3, ApPABP#7 and ApPABP#11 displayed increased binding to rLiPABP in a concentration-dependent manner. Analysis of the data using non-linear regression indicated that aptamers detect rLiPABP in a concentration-dependent manner with Kd = 5.4 ± 1.1 nM for aptamer ApPABP#3, Kd = 6.0 ± 2.6 nM for aptamer ApPABP#7 and Kd = 10.8 ± 2.7 nM for aptamer ApPABP#11.

Next, slot blot assays were performed as above and the results are shown in Fig 3C. As it can be observed, aptamers ApPABP#3, ApPABP#7 and ApPABP#11 showed strong affinity to rLiPABP but not to negative control BSA, thereby indicating their specificity towards the LiPABP target. Interestingly, the results show that only 50 nM of each of the three aptamers are able to detect as few as 6.25 ng of LiPABP.

In order to determinate the lowest number of *L*. *infantum* promastigotes present in a sample detected by ApPABP aptamers, we performed ELONA assays in which total lysates corresponding to increasing amounts of parasites (0–5000) were plated and incubated in the presence of ApPABP#3, ApPABP#7 and ApPABP#11. The results showed that the three aptamers were able to detect LiPABP from 2500 promastigotes in a significant manner (Fig 3D).

### Structural characterization of the aptamers

**Fig 4:**
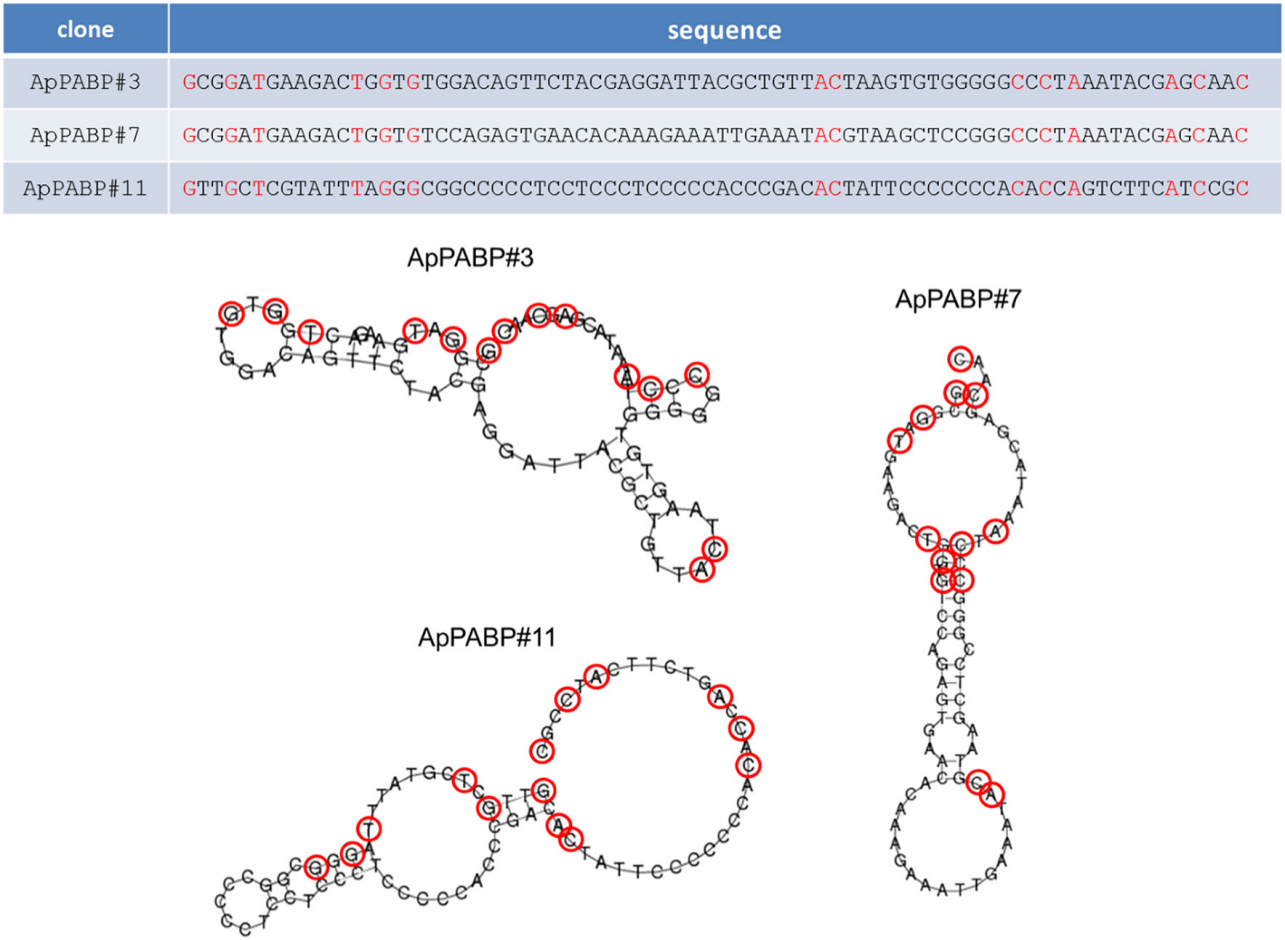
Predicted structural folding of selected ssDNA aptamers for LiPABP. The DNA sequences of ApPABP#3, ApPABP#7 and ApPABP#11 aptamers were analyzed using the program Rapidshare. The resultant predicted secondary structures of the three selected aptamers are shown. The nucleotides labeled in red are those conserved in the sequence of the three aptamers.

To proceed to their structural characterization, the sequences of ApPABP#3, ApPABP#7 and ApPABP#11 were analyzed using the program Rapidshape (http://bibiserv.techfak.uni-bielefeld.de/rapidshapes/) [37] and the potential most stable secondary structures for each aptamer in the same condition that those used during selection are shown in Fig 4. Secondary structure prediction indicates that, taking into account the free energy value, ApPABP#7 would be the more stable aptamer (ΔG = -14.00 kcal/mol), followed by ApPABP#11 (ΔG = -9.30 kcal/mol) and ApPABP#3 (ΔG = -7.40 kcal/mol.). It is interesting to point out that the three aptamers showed non conserved sequences and different secondary structures, suggesting that each of them could bind LiPABP with different affinity and, moreover, they could recognize different “aptatopes” on the protein surface.

### Aptamers inhibit LiPABP-poly (A) complex formation

**Fig 5:**
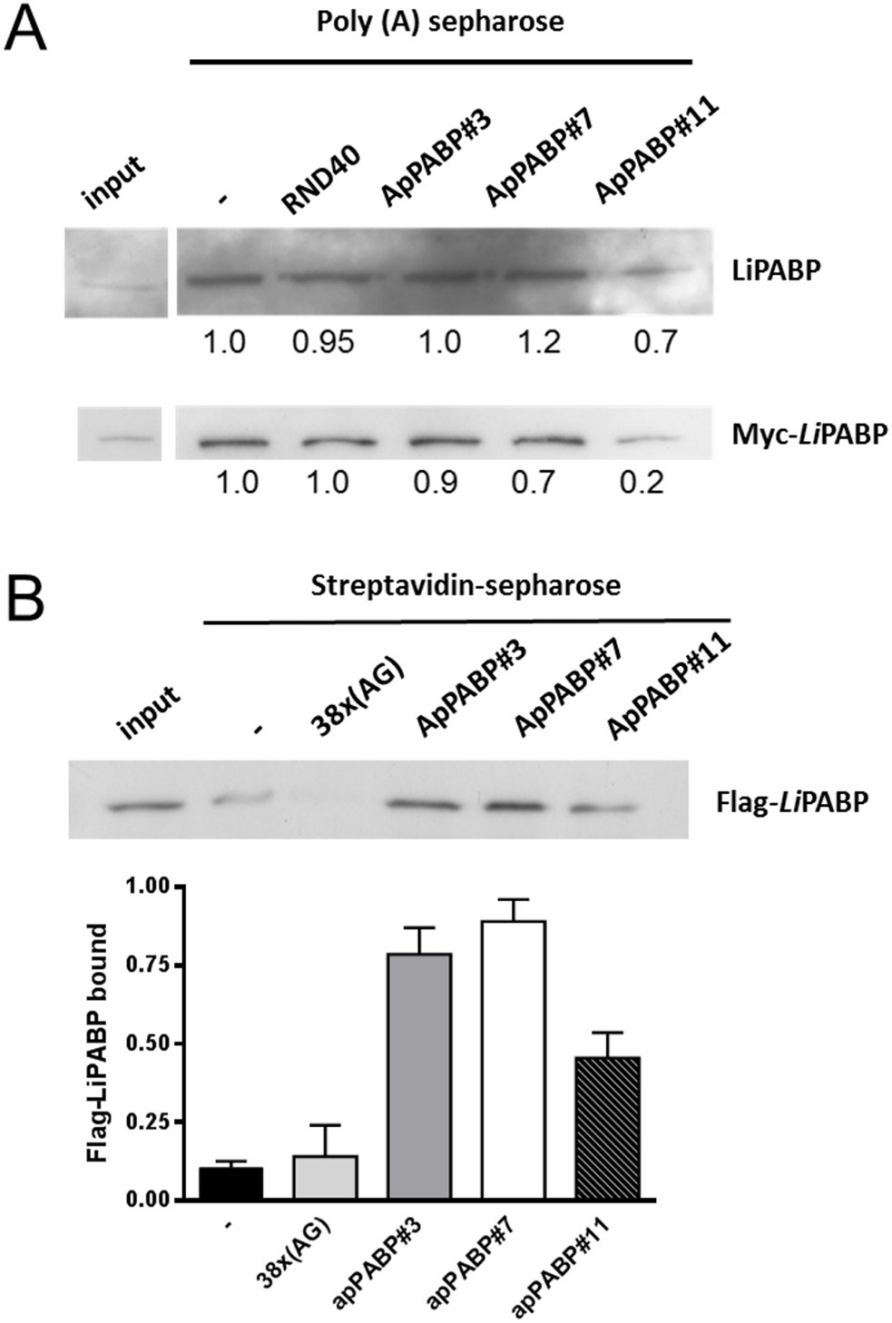
Aptamers inhibit LiPABP-poly (A) complex formation and are capable to pull down LiPABP. (**A**) Representative blots showing the endogenous LiPABP (top) or Myc-LiPABP expressed in HEK293T cells (bottom) bound to poly (A)-sepharose in the presence or not of the different ApPABP aptamers or RND40 are shown. Input corresponding to 50 μg of *L*. *infantum* lysate or 10 μg of lysate from cells expressing Myc-LiPABP was loaded in parallel. Bands were quantified as described in Material and Methods section and the values normalized relative to the LiPABP bound in the absence aptamers. Blot shown is representative of three different experiments. (**B**) Biotin-labeled ApPABP#3, ApPABP#7 and ApPABP#11 or biotinylated 38x(AG) unstructured ssDNA were mixed with 50 μg of Flag-LiPABP-expressing HEK293T cell lysates and the complexes pulled down with streptavidin beads, and Flag-LIPABP protein was detect by immunoblot. A representative blot is shown at the top. Input corresponding to 15 μg of lysate from cells expressing Flag-LiPABP loaded in parallel. Bands were quantified as described in Material and methods section and the values normalized relative to the total Flag-LiPABP in the lysate. Bars represent the mean ± S.E.M. of two different experiments.

The potential effect of aptamers on the interaction of LiPABP with the poly (A) molecule was investigated by a competition assay using the selected aptamers or the naïve RND40 population. As shown in Fig 5A, the amount of LiPABP bound to poly(A)-sepharose was reduced in the presence of ApPABP#11 (30%), while unaffected in the presence of aptamers ApPABP#3 and ApPABP#7 and RND40. To reinforce this result we have also performed the same experiments with lysates from HEK293T cells expressing myc-LiPABP and the band corresponding to the protein bound to poly (A)-sepharose decreased 80% in the presence of ApPABP#11 meanwhile aptamers ApPABP#3 and ApPABP#7 only produce a slight effect. These results indicate that only aptamer ApPABP#11 is able to inhibit LiPABP:poly (A) binding suggesting that this aptamer may recognize the protein at the poly (A) binding site.

### Aptaprecipitation of LiPABP from cell lysates

In order to study whether or not some of the selected aptamers are capable to pull down the recombinant LiPABP, biotinylated aptamers or biotinylated 38x(AG) unstructured ssDNA were mixed with LiPABP-expressing HEK293 cell lysates and the complexes pulled down with streptavidin beads. As it can be observed in Fig 5B, a band corresponding to LiPABP in the three aptamer-bound fractions demonstrated the high affinity of these aptamers for LiPABP. Interestingly, aptamers ApPABP#3 and ApPABP#7 were able to purify LiPABP from lysates with a higher efficiency (above 75%) than ApPABP#11 (below 50%).

## Discussion

Trypanosomatid parasites produce a great deal of chronic diseases affecting hundreds of millions people mainly in underdeveloped countries. Among these parasites, *Leishmania* produces a devastating human disease, leishmaniasis, which severely affects millions of people in Asia, Africa and South America. Although diagnosis and treatment of diseases caused by parasites has undergone major changes due to increased knowledge and technological advances that now allow for more rapid and accurate diagnosis of parasitic diseases, they still need severe improvements. In fact, few major advances in clinical diagnostic testing have been made since introduction of PCR, although new technologies have been investigated [38]. The use of aptamers for clinical diagnosis and therapy is attractive due to their small size, stable folding structure, and economy. There has been rapid advancement in application of such aptamers in disease diagnosis, imaging, and new biomarker discovery [39].

During the last years, we have selected aptamers that specifically recognize *Leishmania* proteins in order to develop detection/therapeutic systems for leishmaniasis. In this paper, we describe the selection and characterization of DNA aptamers that bind LiPABP with high affinity and specificity. In only four rounds of selection, the aptamer pool selected against the LiPABP (SELLiPABP) bind to the target with very high-affinity relative to the naïve RND40 population (Fig 1). From SELLiPABP we have identified three aptamers (ApPABP#3, ApPABP#7 and ApPABP#11) able to bind LiPABP with very high affinity, in the range of the best aptamers previously described [40].

In 2007, Bruno et al [41] reported the development of several DNA aptamers against surface molecules from *Leishmania donovani* promastigotes to be used with diagnostic purposes. Several of these aptamers exhibited very high affinity against *L*. *donovani* and, at lesser extent, *L*. *tropica* showing quantitative binding data in ELONA assays. Most recently, Operational Technologies Corporation (OpTech) has completed a successful Phase I SBIR contract in which 72 new candidates DNA aptamer sequences against *Leishmania major* were developed and screened by ELISA-like plate assay (http://www.sbir.gov/sbirsearch/detail/383773). Taking into account our interest in the development of diagnostic tools for leishmaniasis, we have selected and characterized aptamers against *Leishmania infantum* KMP-11 [18], and histones LiH2A [20, 21] and LiH3 [22] showing affinities and specificities similar than those obtained for *L*. *donovani*. In this paper we demonstrated that high affinity aptamers against LiPABP are able to detect the amount of protein corresponding to only 2500 parasites in a significant manner using ELONA platform. Although this detection limit is far from that desired for a diagnostic system, in our laboratory are working on the development of a more sensible method also based on ELONA, which may be potentially used as a novel diagnostic tool. It is also interesting to note that as far as we know, there are no differences in LiPABP in the promastigote and amastigote stage. In fact, this is an interesting diagnostic advantage due to the possibility of detecting parasite protein released in either forms of *Leishmania*. Some years ago, we reported the development of an electrochemical biosensor based on aptamers that can recognize and specifically detect the presence of *Leishmania infantum* KMP-11 [19]. In that paper, the target protein was conjugated with gold nanoparticles and the complexes electrodeposited on gold screen printed electrodes. Then, complexes were incubated in the presence of digoxigenin-labeled aptamers that specifically recognize LiKMP-11. Using this method with the new aptamers against *L*. *infantum* proteins would be a possibility for the development of a diagnostic test. Additionally, the use of a pool of aptamers recognizing several proteins of the parasites could be a successful diagnostic strategy in different platforms.

From a therapeutic point of view, several strategies have been addressed to affect a parasite organism with aptamers. The first one tries to block the interaction between the parasite and the host. At this respect, aptamers against P. falciparum erythrocyte membrane protein 1 (PfEMP1) [42] and others selected against host-cell matrix receptors (heparin sulphate, fibronectin, laminin and thrombospondin) [43] have been obtained. A second strategy consists in attack the parasite intracellularly and, with this purpose, aptamers targeting heme group which inhibit *P*. *falciparum* growth [44] and others interfering in the intracellular RNA transport in *L*. *tropica* [45] have been selected. In other strategy, aptamers targeting invariant polypeptides in African trypanosomes, which are internalized via receptor, could be used as a specific drug delivery system into the parasite [46]. Finally, RNA aptamers that bind to VSGs are able to recognize different VSG variants and bind to the surface of live trypanosomes to re-direct the immune response of the infected host back to the surface of the parasite independently of the expressed VSG variant. Moreover, these aptamers tethered to an antigenic side group are capable of directing antibodies to the surface of the parasite in vitro providing a new strategy for a therapeutic intervention to fight sleeping sickness [47]. The use of aptamers as therapeutic agents against leishmaniasis diseases has also been studied. Bhattacharyya et al [48] demonstrated that a large number of cytoplasmic tRNAs are imported into the kinetoplast-mitochondrion of *Leishmania* by a receptor-mediated process. In consequence, one important potential role for aptamers in the parasitic diseases treatments would be to interfere in the intracellular RNA transport. Using the SELEX procedure with *L*. *tropica* mitochondria as target, these authors was able to obtain four aptamers containing sequence motifs present in the anticodon arm, the D arm, the V-T region, and acceptor stem of known tRNAs. The aptamers proved to have nanomolar affinities for mitochondria, in the same range as that of natural sequences. These results opened the way to new research lines in mitochondrial mediated tRNAs transport, not only in parasites but also in yeasts and mammals [49]. In a similar way, a specific blocking of the interaction between LiPABP and the poly (A) tail of the mRNA by specific aptamers affecting protein translation in *Leishmania* and could be an interesting therapeutic approach. Our results point out that the three selected aptamers bind LiPABP at different sites because only ApPABP#11 inhibits the LiPABP-poly (A) interaction, while aptamers ApPABP#3 and ApPABP#7 do not have any effect. Taking into account this, we can conclude that aptamer ApPABP#11 could be a potential inhibitor of translation affecting the biology of the parasite. However, this aptamer would have to reach its target into the parasite, who is mainly located into the lysosome of the host cell. In our opinion, aptamers targeting parasite intracellular proteins are not good candidates for leishmaniasis treatment.

It is important to point out that aptamers showing high affinity for the target are not always capable of work in all potential applications. We described in this work the application of aptamer affinity chromatography for one-step purification of LiPABP from a complex mixture. Our results clearly demonstrate that aptamers ApPABP#3 and ApPABP#7 are able to produce a more effective purification of LiPABP from lysates of cells overexpressing the recombinant protein than ApPABP#11, suggesting different binding sites (aptatope) on the protein surface recognized by the aptamers. The feasibility of the method was demonstrated on *L*. *infantum* PABP overexpressed in HEK293 cells, but we anticipate that it could be used for any other protein for which aptamer development is possible.

In summary, we have generated a population of ssDNA aptamers that bind with high specificity to *L*. *infantum* PABP antigen and do not bind to other proteins of the parasite. The current work demonstrates that aptamers can be used for detection of specific targets in an ELONA format. It is important to point out that aptamers have a great potential to circumvent limitations associated with antibodies. Although the antibody detection system is the gold standard for protein detection and identification, the data presented in this study strongly supports, due to easier and lower cost of their production, the feasibility of the aptamer-based diagnostic system. In conclusion, it is reasonable to expect that the ELONA platform using aptamers will stimulate the development of other detection systems in which antibodies have been previously used. Aptamer affecting the binding of LiPABP to the poly (A) tail is a potential candidate for leishmaniasis therapeutic. Furthermore, some of the developed aptamers can be used in the affinity purification of the target protein from cell lysates. Considering the extreme complexity of cell lysate, aptaprecipitation can be extended to potentially any biological mixture such as blood, urine and others. This method facilitates the faster recovering of recombinant proteins in pure form for their structural and functional studies as well as for other biomedical applications.
